# On the Design of Social Robots Using Sheaf Theory and Smart Contracts

**DOI:** 10.3389/frobt.2021.559380

**Published:** 2021-08-18

**Authors:** Renita Murimi

**Affiliations:** University of Dallas, Gupta College of Business, Irving, TX, United States

**Keywords:** blockchain, robotics, smart contracts, sheaf theory, imperfect information, irrationality

## Abstract

The incorporation of robots in the social fabric of our society has taken giant leaps, enabled by advances in artificial intelligence and big data. As these robots become increasingly adept at parsing through enormous datasets and making decisions where humans fall short, a significant challenge lies in the analysis of robot behavior. Capturing interactions between robots, humans and IoT devices in traditional structures such as graphs poses challenges in the storage and analysis of large data sets in dense graphs generated by frequent activities. This paper proposes a framework that uses the blockchain for the storage of robotic interactions, and the use of sheaf theory for analysis of these interactions. Applications of our framework for social robots and swarm robots incorporating imperfect information and irrationality on the blockchain sheaf are proposed. This work shows the application of such a framework for various blockchain applications on the spectrum of human-robot interaction, and identifies key challenges that arise as a result of using the blockchain for robotic applications.

## Introduction

A common problem in decision-making is that of imperfect information. Imperfect information limits the size of the potential action space for players, and the consequences of a player’s actions sets off a domino effect when confronted by the actions of other players who are also operating under imperfect information. Imperfect information is one of several factors that propel human actors to the use of biases in decision-making. Biases simplify decision making, since an actor needs only to revert to the previously used set of actions or decision processes when dealing with the current problem. The impact of these biases is felt as shortcuts in decision-making in our everyday lives influencing our choices in areas such as purchasing, diets, friendships and social network formation (Hilbert, 2012). Pioneered by Daniel Kahneman and Amos Tversky (Kahneman and Tversky, 1986), the role of biases in decision-making exposed a puzzling trait of the human actor in the form of the “irrational man”. The irrational actor makes sub-optimal choices, supported by perceptions of a greater utility from the outcome of her actions. In the presence of imperfect information, the irrational actor resorts to biases and in the company of like actors, these biases have the potential to be amplified through collective herding or swarming behavior (Beni, 2004). This collective behavior of irrational actors has defied traditional theories of utility derivation, which hitherto assumed rational actors making optimal choices.

While technology has ushered in the era of artificial intelligence, machine learning and massive datasets, these advances have also tilted the scale of rationality in favor of machines that can parse through large action spaces which are far beyond the reach of human cognition. The robotic versions of Jeopardy (Ferrucci et al., 2010), chess (Knight, 2017) and AlphaGo (Silver et al., 2017) have demonstrated the power of modern computational machines that evaluate all possible outcomes of an action undertaken by the machine and the opposing player. In doing this, these robots incorporate past, current and future action spaces, thereby exercising a superior breadth of decision-making over the human player. Compared to the robot, the human actor typically has a limited recall of the past, is short-sighted in the current and is usually myopic about the future. In essence, these games comprise of two unevenly-skilled players. On the one hand is a robot with perfect information in a niche application (the AlphaGo robot would not be expected to skillfully pick up a block). On the other hand, is a human with imperfect information in a range of applications, and is ill-suited to match the prowess of the machine with perfect information. In all of the three games (AlphaGo, Jeopardy and chess), the speed at which the machines dominated the game continues to improve. Simultaneously, the chasm of informational asymmetry widens as ethicists and engineers simultaneously marvel at the feats of the machine and groan at the rate at which the human succumbed to the machine. Human heuristics, although not limitations, are inadequate at matching the niche-specific abilities of the robots.

The algorithms that power these robots tend to reduce or eliminate two crucial factors in decision-making: imperfect information and irrationality. While it is tempting to think that imperfect information and irrationality are two sides of the same coin, observations tend to suggest otherwise. For example, in the case of a traffic crossing, perfect information offers a pedestrian complete view of traffic and tells a pedestrian to cross at the appropriate prompts. However, an irrational pedestrian may choose to defy this perfect information and choose to walk into oncoming traffic with disastrous consequences. The algorithms that comb through massive datasets of demographic information, voting polls, SAT results, credit histories and retail transactions (O’Neil, 2016) have a common two-fold objective: reducing imperfect information by detecting patterns and choosing rational decisions tailored for each pattern of behavior in the data. This serves us well when the machines help us detect cancer faster, correlate income to majors and in general, predict the future when our limited purview of what lies ahead is hindered by our inability to remember everything and find patterns fast. However, what happens when these machines are no longer used to help us but are finding their spot in our society as co-equals?

This question is no longer in the realm of a sci-fi future. Machines powered by artificial intelligence are our talking assistants, entertaining our children and keeping the company of elders. Drones have their own social media pages, and it is not far-fetched to think of a society that is inhabited by humans, robots, software bots and IoT devices collocated in both physical and virtual spaces. For example, it might be that we meet a robot for coffee in a restaurant, to which our self-driving car takes us. In such a scenario, it is hardly comforting to think that certain entities, such as robots are far more superior to humans in every instance of an application. Thus, who would want to play with a robot that always wins? Or to socialize with a robot that knows everything and has an answer to every question? Much of human interaction is bound by the glue of empathy, where like-minded and likely-abled individuals bond over common interests and challenges. As more and more robots are employed for social interaction, singular dominance and expertise on the part of the robot are not only downright undesirable, but also detrimental to the adoptability and applicability of robots in social situations.

The use of blockchain technology has inspired the development of frameworks in diverse disciplines, including social robotics. In Marsal-Llacuna (2018), the author proposes a framework called the Future Living Framework concerning blockchain for cities of the future. Another blockchain framework was proposed in Cebe et al. (2018). Here, the authors propose the use of a blockchain framework to resolve potential disputes related to vehicle accidents that might arise from the use of driverless cars. Blockchain’s immutable ledgers can be used as an authoritative source of information for vehicular forensics. The addition of smart contracts to the blockchain is especially appealing to an ecosystem inhabited by robots. Smart contracts are digital contracts that are added in code to the blockchain. Work in (Strobel, Ferrer, and Dorigo, 2020) presents the findings of a swarm robotics application, where the robots used blockchain-based smart contracts to estimate the number of black tiles in an environment covered with black and white tiles. These codified contracts are executed only after the fulfilment of certain clauses, thereby eliminating the need for intermediate entities to provide oversight and regulatory compliance. Creating robotic applications with smart contracts that reside on the blockchain can enable the inclusion of tuning parameters such as varying degrees of imperfect information and irrationality. For example, a robot that interacts with a child in a gamified form of learning might behave differently than in a corporate environment where it is training employees on job functions. In this scenario, the parameters of imperfect information and irrationality might be differently tuned to adhere to each of the learning environments.

From a graph-theoretic perspective, smart contracts facilitate the creation of additional edges on a graph upon fulfilment of underlying clauses. Again, this creates dense graphs and has the potential of launching a cascading effect of creating more edges as the underlying conditions are satisfied. The analysis of such graphs with dense structures poses a distinct computational challenge, where increasing size of the network with heterogeneous data collection and processing needs creates dense graphs that cannot be efficiently analyzed for patterns. This scenario is further complicated by the potential for smart contracts to bind with billions of IoT devices, robots, humans and software bots that will create large amounts of graph data.

In this paper, we propose a robot-learning framework for social robotics that is built upon two important qualities that make human actors “human”. Specifically, our framework utilizes imperfect information and irrationality as foundational aspects of the robot-learning mechanism. The imperfect information and irrationality can be tuned to different levels depending on the application, thus resulting in dynamic action spaces. Since dynamic state spaces impose a significant computational burden with current graph-theoretic tools, we propose the use of topology theory in the analysis of these dynamic spaces. Specifically, we propose the use of sheaf theory to derive important features about the shape of the action spaces created by human-robot and robot-robot interaction. Finally, these features are stored on the blockchain, thus harnessing the blockchain’s ability to store data in a distributed, trusted immutable manner.

## Related Work

Algebraic topology is finding applications in various domains such as robotics (Farber, 2006), information theory (Robinson, 2013), circuit design (Robinson, 2012) and network science (Mansourbeigi, 2017). In (Sizemore et al., 2019), the authors explored topological data analysis to study the shape of genomes and synapses. They showed how algebraic topology was able to visualize cavities in neural structures. These cavities might arise due to the presence of correlations in certain regions of the brain and could be indicative of disease. Topological data analysis has also been used to study social dynamics (Maletic and Milan, 2014). Here, the authors study the formation of consensus through the evolution of opinion maps in social networks. Using a topological feature known as simplicial complex, this work studied the evolution of the opinion spaces as a function of number of agents, number of opinions and the dynamics underlying the emergence of the most popular opinion. The literature most closely related to our work is in (Meldman-Floch, 2018), where the author models a block as a sheaf and develops a theory for distributed consensus protocols. Human-robot interactions captured on a blockchain with smart contracts have been studied in (Cardenas and Kim, 2018) where the authors propose a case study for a traveling robot that can enter into financial agreements stored on the blockchain in exchange for assistance in traveling the world. Work in Robinson (2012) has proposed a sheaf-based framework for understanding the behavioral properties of a logic circuits, and emphasized the role of a sheaf-based analytical framework in uncovering hazardous conditions such as timing-related race behavior between elements in a combinational circuit. The ability of sheaf-theoretic frameworks to decipher global information from local information have led to a diversity of application such as those that have been further proposed to model concurrent processes in distributed systems (Malcolm, 2009), semantics for object-oriented programming languages (Cirstea, 1995) and representations of information systems (Sagar and Kishore, 2019).

The applications of sheaf theory to modeling of robotic systems is an emerging domain. Modeling the interactions of system components using sheaf theory has been studied in Sofronie (1996). Here the author showed that interacting system components can be modeled using geometric logic, with states, parallel actions, transitions and behaviors being represented as topos of sheaves. A specific application of sheaf theory to robotics has been described in Pfalzgraf and Stokkermans (1995), where the notion of fibered bundles or sheaves is applied to cooperating agents or robots. The local-global properties inherent to sheaves were shown to be useful in modeling the communication and interaction between cooperating robots. The study of event-based systems using sheaves is described in Zardini et al. (2020), where events belonging to a system denoted by time stamps and the transition between these events can be depicted in a topological space. Examples of event-based systems that are analyzed in this paper include the representation of background on event cameras and robotic system description. Work in Chemello and Sossai (2006) applied sheaf theoretic approaches to the problem of uncertainty in data fusion. The mechanism of “gluing” in sheaves has been applied to combining data from different sources with different semantics, where the authors showed that the higher-level abstractions offered in sheaf theory can be useful in applications such as data fusion modeling. Another application of sheaf theory for modeling robot control interaction is in Nakamura (2015), where the author studies the stabilization and control of two-wheeled mobile robots using sheaves.

## A Brief Overview of Social Robots

The notion of employing machines for social interaction with a fusion of human, animal or machine-like capabilities is not new–earliest mentions of social robots extend back to roughly a century ago with the development of the Elektro robot, the proposition of Asimov’s three laws of robotics and the bold vision of the Turing test. In Bartneck and Forlizzi (2004), the authors define social robots as “autonomous or semi-autonomous robot that interacts and communicates with humans by following the behavioral norms expected by the people with whom the robot is intended to interact”. Thus, social robots are distinguished by their ability to communicate and interact with humans, and not just with other robots. The authors propose a five-pronged framework for studying social robots–form (shape, behavior and behavior), modality (number of communication channels), display of social norms, autonomy and interactivity. Further, the authors provide a set of guidelines for the design of social robots. These guidelines for human-robot interaction (HRI) suggest that the robot’s design that matches its abilities, possess the ability to engage in human-robot communication, and the ability to mimic human social norms.

Next, we profile some of the widely known modern social robots. The goal of this section is two-fold. First, this section draws attention to the prolific field of social robots and their evolving capabilities. Secondly, it highlights the nature of interactions between social robots and humans that warrant the need for novel, efficient frameworks to store, process and analyze the interaction data between humans and social robots.

PARO is a social robot which is a baby harp seal robot designed as play therapy for older adults with dementia (Hung et al. (2019), (Wada et al., 2010). In Hung et al. (2019), the authors describe the use of a social robot called PARO in a geriatric dementia unit. Here, the authors address how psychosocial approaches employing social robots like PARO can contribute to personhood-centered care. Kitwood (1997) defined personhood as being linked to the five fundamental human psychosocial needs (comfort, attachment, occupation, identity and inclusion), with love being at the core. The study in this paper described how PARO was used in diverse settings such as assisting patients during medical procedure to ease discomfort, and providing companionship as with a buddy. In particular, patients interacted with PARO *via* smiling, kissing, hugging and talking. Each of the emotional bonding moments with PARO was recorded, and patients treated PARO with respect and reciprocated social norms as one would with a friend. It is important to note that patients were informed that PARO was a robot, still the patients were able to establish social connection with PARO by caring for it and treating it as a buddy. The introduction of PARO to the geriatric dementia unit was shown to improve relational care and provide support for each of the five elements of personhood.

In Bharatharaj et al. (2017), the authors develop a parrot-inspired robot, KiliRo, for teaching children with autism spectrum disorder and to improve their social interaction abilities. Researchers would teach KiliRo letters, numbers and human recognition in the presence of children, who would then be motivated to participate in the learning process upon watching KiliRo interact with the researcher. The acceptance, likability and interaction interest of the children toward KiliRo, in addition to their progress in learning and social interactions were measured throughout the study. Further, the responses of parents to the use of KiliRo as a therapy for their children’s behavior were recorded through questionnaires and interviews. It was found that KiliRo was well-received by the children, parents and physicians. Facial recognition software employed in the study showed that the children were not afraid of Kiliro and were happy to interact with it.

Another application of a social robot for robot-assisted therapy (RAT) with children is in Saldien et al. (2010), where the authors describe a robotic user interface application for a social robot called Probo that interacts with hospitalized children. Designed to look like an anthropomorphic elephant, the researchers provided the children an elaborate story of Probo’s family tree starting in the Ice Age and drifting around the earth to provide joy to children. Probo is designed to have a fully actuated head with a variety of motions, and was able to use vision, touch and audio stimuli to interact with children. Probo is also capable of displaying multiple facial expressions and emotional cues. The goal of this study was to evaluate the effectiveness of Probo in displaying emotions through facial expressions in its interactions with children and adults. The study showed that certain emotions (e.g. happy) were recognized more easily than others (e.g. fear).

Sony’s robotic dog, Aibo, is one of the widely known social robots. Work in Friedman et al. (2003) studies human perceptions of Aibo through analyses of established online forums dedicated to Aibo. Their analysis showed that approximately half of the discussions mentioned in these forums ascribed life-like abilities to Aibo. These discussions contained language that referred to Aibo as having feelings and emotions, which in turn elicited behavior from the participants akin to interacting with a pet. Aibo, in turn, is capable of reciprocating with a wide range of pet-like behaviors that range from mimicking the actions of its human companions, playing with a ball, walking, sitting and emoting.

Work in Kuchenbrandt et al. (2011) analyzed human perceptions of the social robot called Nao. Here, the authors sought to discover how humans treated robots who were part of their group *versus* robots who were external to their group. Human participants were asked about their willingness to interact with Nao by talking to it, getting to know it more closely, liking it and eventually buying it. Their findings showed that humans were more likely to engage with Nao and anthropomorphize it if they perceived the robot to be a part of their group. Work in (Scheef et al., 2002) describes the design of Sparky, a social robot that can display various facial expressions and up to nine different emotional states. The goal of this work was to understand the kinds of responses that Sparky’s emotional reactions elicited among humans. Unlike Aibo which is an autonomous social robot, Sparky is teleoperated by a human controller. Here, the authors found that Sparky elicited the most response when it was presented with opportunities for eye-eye contact with humans. Another application of social robots is described in Garrell and Sanfeliu (2012) for social-navigation assistants, Tibi and Dabo, in crowded urban environments. These twin mobile service robots were designed to accompany humans on their walks in urban environments, thus requiring them to be “aware” and react appropriately to the presence of other humans and obstacles in their environments. Other applications of social robots have been studied in Peter et al. (2019), where the authors analyze social robots that interact with children in an Internet of Toys paradigm (Mascheroni and Holloway, 2019).

While there has been considerable research done in separate domains of ethical robot behavior, robot learning and robot interaction, the disparate nature of work done in machine learning, graph theory and robot ethics poses distinct challenges in developing robotic applications that enable efficient robot integration in human societies. Research in Tatsukawa et al. (2019), Chatila et al. (2019) has pointed to the need for robots to be self-aware and environment-aware, thus implying the need for robots to develop human-like abilities for more meaningful interaction with humans. Our work proposes a framework for robotics applications on a blockchain that is represented using a sheaf-theoretical framework. Incorporating smart contracts into the sheaf-theoretic representation of the blockchain allows for tunable robots–ones that can be attuned to the dynamics of the environments that the robots inhabit. Our work emphasizes two key parameters for the socialization of robots–imperfect information and irrationality that have implications in ethics, learning, accountability and social interactions. This paper seeks to develop a foundational framework that incorporates robotics applications on a blockchain with provisions for incorporating a variety of learning models, rewards, ethical constructs and social integration incentives. The use of a blockchain enables utilization of its distributed, secure, immutable ledger properties for storing attributes pertinent to robot learning, interaction and socialization. Visualizing the blockchain as a sheaf using tools from algebraic topology allows for faster computational models, analysis of hidden structures and patterns in the interaction data as well as integration with cross-domain applications. The rest of this paper develops our model, presents examples of applications and discusses challenges and limitations.

## A Unifying Framework for Blockchain, Robotics and Sheaves

In this section, we describe our approach to incorporating robotics applications on the blockchain that will focus on modeling the blockchain as a topological structure of sheaves. We build upon work in Zardini et al. (2020) for a quick overview of underlying topics in category theory, and refer the reader to Riehl (2017) for a detailed exposition of category theory.

### Blockchains in Algebraic Topology

Consider blockchain from the viewpoint of combinatorial algebraic topology (Goldblatt, 2014). This viewpoint offers a suitable framework for studying blockchain with the set of all nodes as the node set and the set of all transactions as the transaction set. In conventional graph theory, nodes are vertices and an edge represents a transaction between two nodes. However, combinatorial algebraic topology lets us represent many different aspects of transactions. Transactions may originate from multiple nodes and may be destined toward multiple nodes. Transactions may also possess certain domain-specific properties such as recurring bill payments, duplications of key aspects of records, and device information of IoT devices and may be bound by smart contracts.

Combinatorial algebraic topology allows such interactions to be captured through the construction of simplices. An n-simplex is the convex hull of its n+1 vertices. Thus, a 1-simplex is an edge, a 2-simplex is a triangle, a 3-simplex is a tetrahedron and so on. A simplex is further characterized by the presence of faces. If A is a 1-simplex, its faces are the edges $A_{0}$ and $A_{1}.$ On a graph, a 1-simplex represents an edge which is a transaction on the blockchain involving two nodes.

**FIGURE 1:**
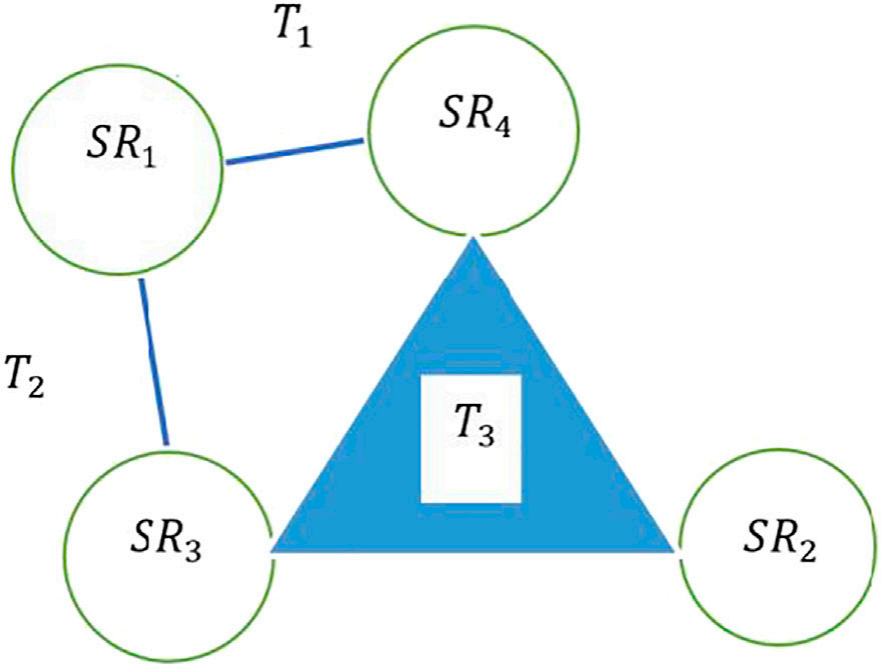
A representation of task collaboration among sensors of a robot using simplicial complexes.

A simplicial complex represents a mechanism to visualize complex data sets with multiple dimensions and is formed by attaching individual simplices. While traditional graph theory uses the foundational model of nodes and edges to represent unidimensional bidimensional connections between data points, data sets that contain multidimensional connections need modeling mechanisms that are capable of representing these complex interactions. Constructs from topological theory such as simplicial complexes provide tools to represent combinatorial representations of data points. For example, consider an interaction between four sensors of a single social robot $SR_{1},\;SR_{2},\;SR_{3}$, and $SR_{4}$ as shown in Figure 1. Each edge represents an interaction between the sensors at the endpoints of the edge, denoting collaboration between two sensors on a given task. Thus, $SR_{1}-SR_{4}$ denotes collaboration between sensors $SR_{1}$ and $SR_{4}$ on task $T_{1}.\;$Simultaneously, the simplicial complex depicted in Figure 1 offers the capability to represent collaboration between three nodes on task $T_{3}$, as demoted by the shaded face $SR_{2},SR_{3}$, and. $SR_{4}.$

Presenting blockchain transactions in the context of combinatorial algebraic topology offers many advantages. The scale of blockchain and robotics is poised to increase, as more and more applications are envisioned to use blockchain for its distributed, immutable foundational properties. Scalability in blockchain has been addressed in Bano et al. (2017), where the authors describe the patterns of collective leadership and sharding in blockchain as mechanisms to delegate consensus and distribute accountability in favor of speed and scalability. Storing all these application-specific data points on the blockchain poses a significant challenge for blockchain analysis. For example, consider the problem of fraud detection. Fraud detection relies heavily on spatiotemporal patterns to reveal information about (Abdallah et al., 2016) fraud. By using conventional graph theory to extract this information from the blockchain, the analyst will have to sift through graph representations of transactions on the network. Parsing through graph-data is computationally expensive and may not readily reveal hidden properties of the transactions. Specifically, as transactions become complex, it becomes challenging to visualize higher-dimensional data. For example, social network data stored on the blockchain has the potential to resemble dense point clouds due to the structure, scope and frequency of user activities on the network. Dense point clouds can hide valuable topological features such as clusters, voids, cuts, tunnels, pieces and presence of curved surfaces (manifolds). While one might argue that approaches such as principal component analysis (PCA) might reveal topological features (Jolliffe and Cadima, 2016) and thus expose interrelationship between data points, this method fails to accurately detect curved surfaces in the data.

Representation of a complex structure such as blockchain from a topological perspective enables us to create simplicial complexes that approximate the graph of the blockchain. Since blockchain transactions depend on peer effort to mine and verify transactions, this activity along with the original transaction can be represented as simplices that overlap through their shared vertices. The overlap of shared vertices in simplicial complexes can be used to determine the structure of the blockchain with respect to features such as eccentricity and vertex significance (Maletic and Milan, 2014). Here, the authors measure the eccentricity as the integrity or individuality of the simplex. An eccentricity of zero indicates complete integration of the simplex, i.e. the simplex is the face of another simplex. Conversely, an eccentricity of one indicates a completely disintegrated simplex that does not share faces with other simplexes. Vertex significance extends this concept by measuring the scope of integration of a simplex. Vertex significance measures how many other simplexes the vertex is a part of; thus, a high value of vertex significance indicates a vertex that is a part of many simplexes. These features in *n*-dimensions can be readily captured by a topological approach to studying the blockchain thus revealing information about clusters, hubs, influencers, anomalies and fraud. Techniques such as the Vietoris-Rips complex for simplicial complexes can further aid in analysis by providing means to de-noise data using thresholding. For a detailed treatment of Vietoris-Rips complex, the reader is referred to Curry (2014).

### Construction of the Blockchain Sheaf

Consider the graph G of nodes and edges. A sheaf F on G consists of a vector space of vertices F(v) for each v, a vector space of edges F(e) for each e, and a linear transformation map F(v)→F(e) for each incident vertex-edge pair. The vector spaces F(v) and F(e) are stalks of F over v or e, bound together by linear transformation maps, also known as restriction maps. The restriction maps provide tools for local consistency verification. For example, the Proof-of-Work (PoW) required to verify a transaction on the blockchain can be viewed as the local consistency checking function that verifies a link between the current transaction and previous transaction. This sequence of PoW consistency functions can be pieced along all the way to the first transaction, thus providing a central spine or stalk pieced together by individual transactions, or germs of the stalk as in sheaf theory. This same analogy can be applied to a higher scale for depicting relationships between blocks. Depicting the blockchain as sheaves allows for generalizations across scales from individual transactions to blocks. As we shall see later in this section, sheaves enable depiction of higher dimensional data on the blockchain.

**FIGURE 2:**
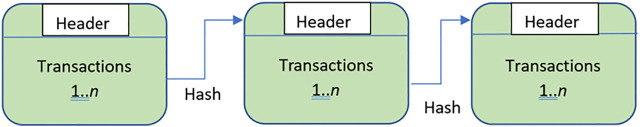
Abstract representation of the blockchain. Each block contains transactions, and a block’s hash is included in the header of the successive block.

**FIGURE 3:**
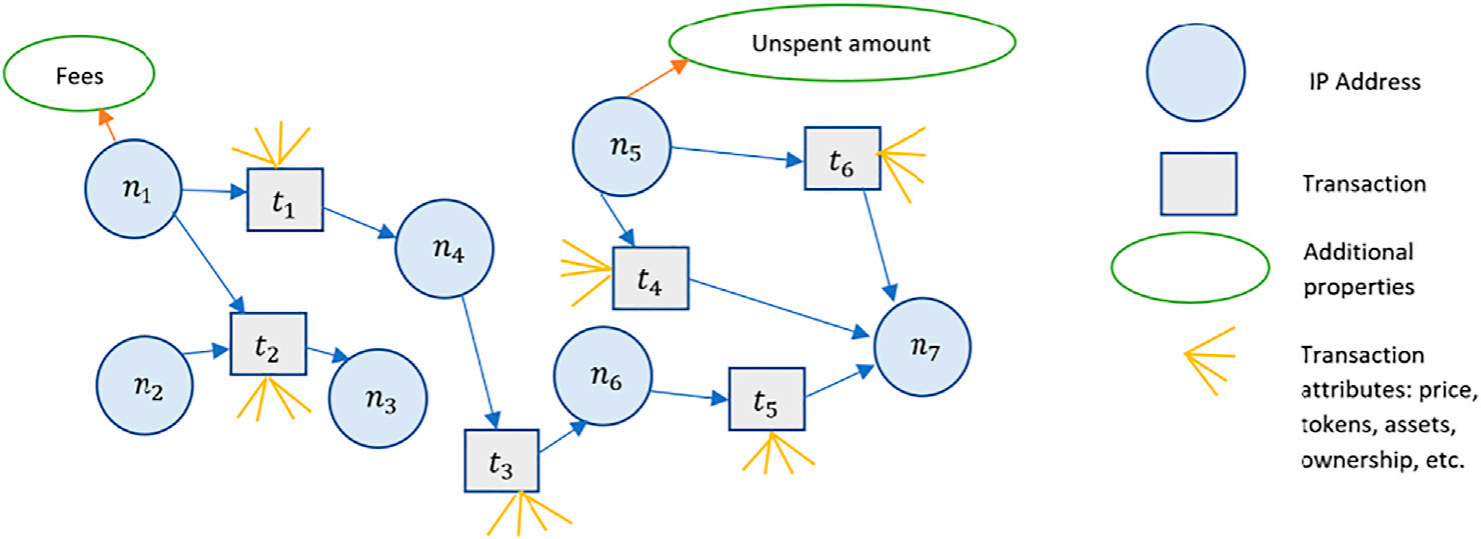
Graph representation of the blockchain. IP addresses, represented as nodes $n_{1}-n_{7}$ engage in transactions $t_{1}-t_{6}.$ Transactions attributes are shown.

**FIGURE 4:**
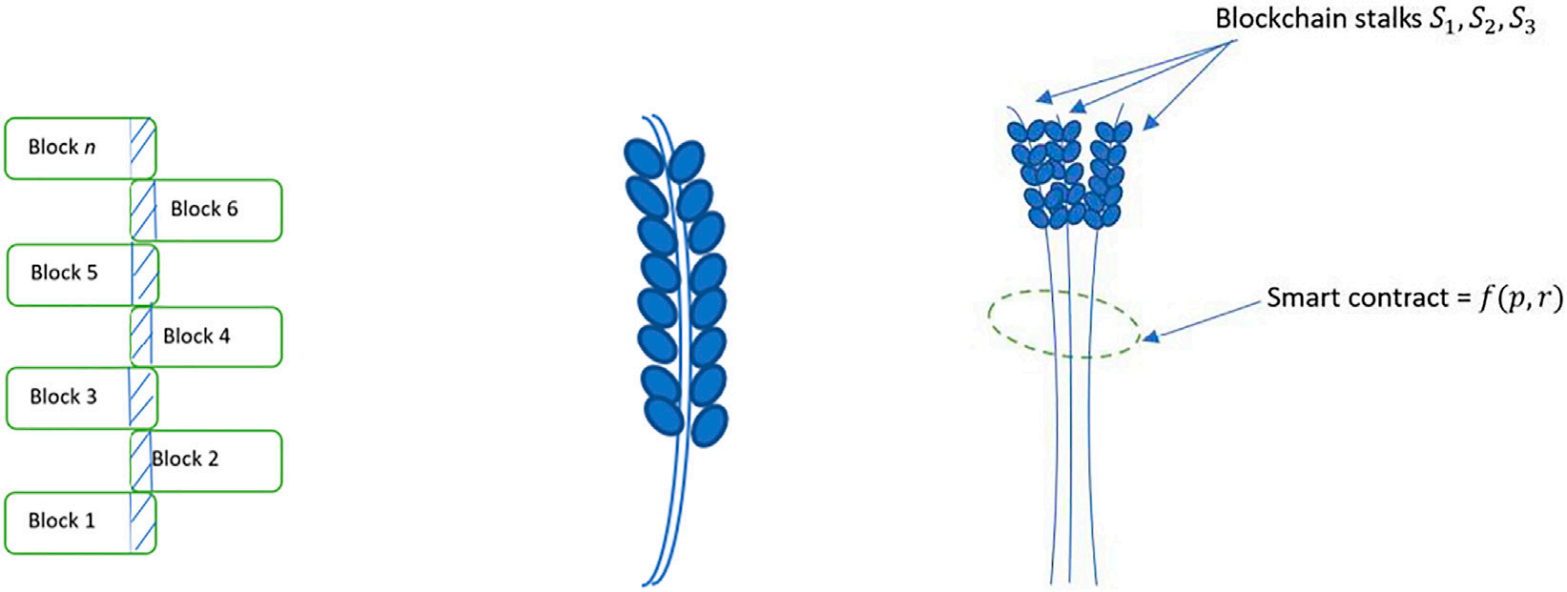
An analogy describing stalks and blocks. Left: Blockchain as a stalk. The shaded portion represents the hash functions that link blocks to their successors and predecessors, thus forming a central spine around which transactions are linked. Middle: An individual stalk with seeds. Right: A sheaf of blockchain stalks bound by a smart contract tuned with imperfect information (p) and irrationality (r).

Figure 2 shows an abstract representation of the blockchain. Figure 3 shows a graph representation of the blockchain in Figure 2. We see that the graph representation adequately captures the nodes, transactions (edges) and transaction amounts (edge weights). Additionally, directionality can be incorporated to identify the sender and receiver of a transaction. However, as we add additional attributes to the vertices or the edges, the graph tends to offer an inadequate representation of the richness of features on the blockchain. Figure 4 shows the blockchain as a stalk, where individual transactions in a block represent germs in the stalk. This representation can be abstracted to include multiple stalks in a sheaf. Increasing the dimensions of the vertex and edge stalks can be used to represent additional dimensions of the blockchain. For example, Figure 4 shows a blockchain sheaf of three stalks. The smart contracts are executed only upon fulfilment of underlying conditions that bind entities with information on the blockchain sheaf. Thus, entities with limited information and high irrationality are able to co-exist with other entities possessing high information and limited rationality. These entities might represent software applications, autonomous robots, or humans and their interactions are stored as blockchain sheaves.

**FIGURE 5:**
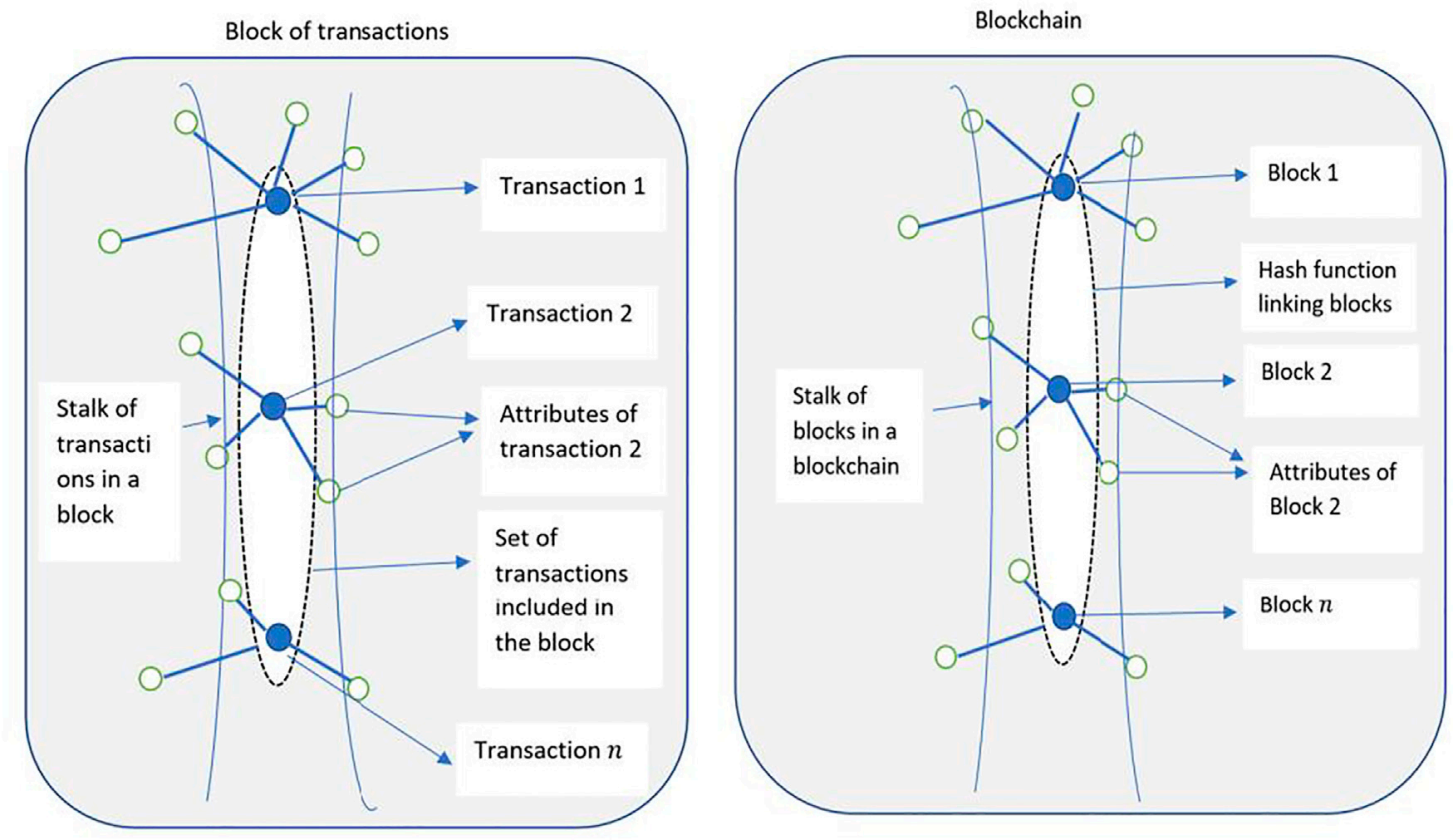
Left: A block of transactions represented as a stalk. Individual transactions contain varying attributes. Right: A blockchain of n blocks represented as a stalk. Block-level and transaction-level attributes can be adequately represented in the sheaf-theoretic model.

Higher dimensional attributes of the blockchain graph can be represented as shown in Figure 5. Figure 5 shows the blockchain from Figure 2 along with its higher dimensional attributes represented as multiple connections emerging from a single nod. Such representations are useful in finding similarities in the topology to identify like nodes, clusters and network partitions.

**FIGURE 6:**
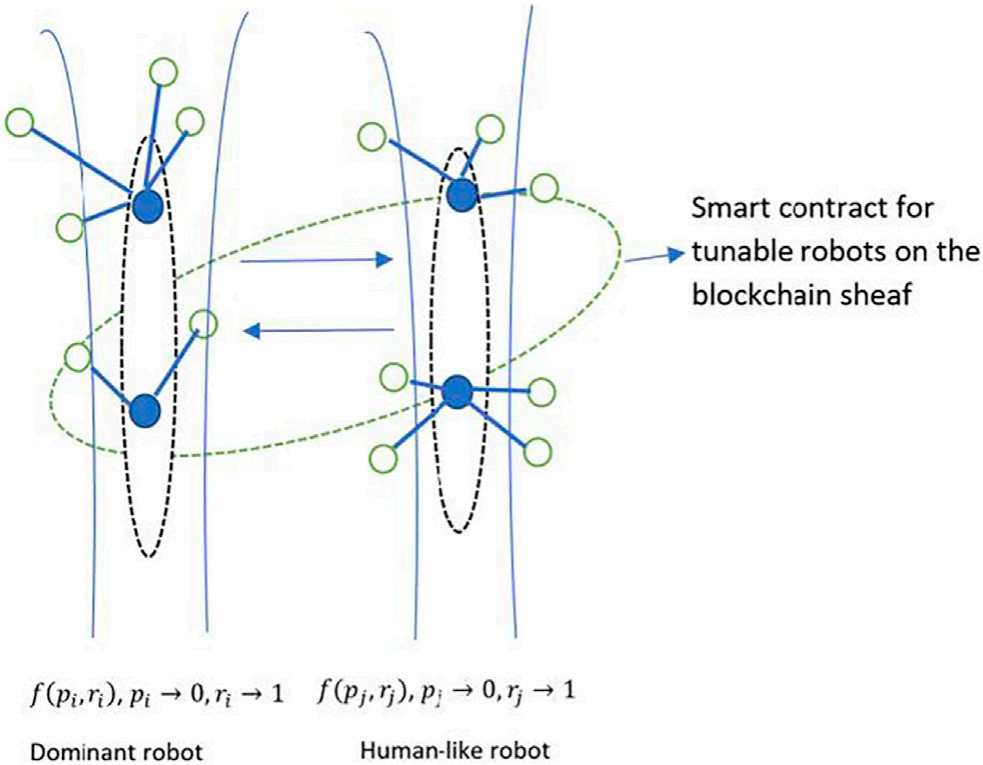
Stalks representing social robots. Left: A controller robot. Right: A lower-level robot.

**FIGURE 7:**
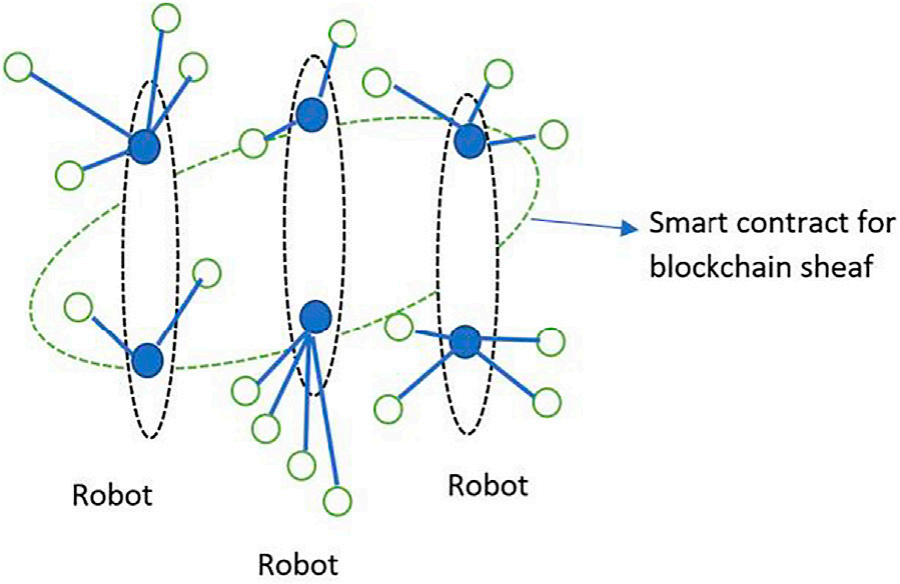
Blockchain sheaf of multiple robot interactions.

Another example of blockchain sheaves is shown in Figure 6 for a social robotics application. The two sheaves represent the imperfect information and irrationality constraints on the blockchain. The blockchain hosts a social robotics application, where multiple robots with limited functionality cooperate to complete a higher-level complex task such as a pursuit-evasion game. The sheaf on the left depicting a dominant robot belongs to a controller robot which possesses almost perfect information and rationality. The sheaf on the right represents a lesser-abled robot that possesses higher levels of imperfect information and irrationality than the controller robot. Thus, the sheaves $S_{1}$ and $S_{2}$ might represent robot consensus over target locations. The consensus could be arrived at using information gathered by individual robots about the target’s location. Smart contracts for blockchain sheaves enable role swapping to upskill existing robots’ functionality, or to allow for load balancing for networks with sensors of different capabilities, or for robots with varying skills (Figure 7). Additionally, tools from algebraic topology such as persistent homology (Edelsbrunner and Mozorov, 2012) can be used to detect robustness of the components of the motion graphs.

### Applications

In this section, we envision applications of our model for swarm robotic and social robotic ecosystems on the blockchain of sheaves bound by smart contracts.

Swarm robotics: Swarm robotics harness concepts in emergent behavior from formation of swarms in nature, such as swarms of bees that rely on collective intelligence to accomplish complex tasks that might be difficult for the single organism, but through synchronization, delegation and feedback are accomplished by groups of organisms. The ideas borrowed from swarm formations in nature have influenced several advances in robotics (Brambilla et al., 2013) by relying on tiny, cost-effective robots that are able to communicate with each other and coordinate their activities to accomplish complex tasks such as mining and foraging operations, UAVs, disaster rescue missions and military applications. The use of blockchain for swarm robotics has been explored in (Ferrer, 2018), where the authors propose the use of blockchain to provide better security, enhanced distributed decision-making, and the ability to coordinate in an environment populated by heterogeneous robots. Thus, individual robot transactions are stored in blocks that link to form a distributed ledger. Our proposed model of imperfect information and irrationality can be encoded in sheaves and smart contracts for swarm robotics, and can be robustly applied to several scenarios, as shown in Figure 7. For example, swarm robots in a mining operation may utilize multiple kinds of robots for detecting motion, sound, heat, carbon dioxide or other parameters. Each of these robots operates under imperfect information of the environment and the ad hoc network. With time, the imperfect information parameter is gradually refined as more and more sensors from the robots gather information about the environment and each other. The irrationality parameter of the robot prevents the robot from jumping to conclusions about the environment and serves as a nudge to rely on collective intelligence of the ad hoc network.

As the robots communicate with each other forming ad hoc networks, each sensing and communication activity is stored on the blockchain and can be viewed as a germ, which links to other germs to form stalks. The linkages in forming stalks may be based on any scheme - the class of robots (heat detector), operational category (high-altitude motion detection), high-level functions (data analysis) or network maintenance (signaling for additional robots to a particular site in the event of viable leads). Each of these stalks coexists with other stalks formed from various schematic configurations, allowing for redundancy in operations. This sheaf of co-existing stalks is bound by smart contracts, which allow for just-in-time execution of higher-level tasks. Sheaves enable compact representations of the decision spaces found in swarm robotics applications, since the local and global properties of the sheaf impose fewer computational resources than a graph-theoretic approach. This enables a richer representation of the blockchain, enabling a deeper understanding of features such as persistence (Edelsbrunner and Mozorov, 2012), influence and clustering.

Social robots: Social robots are an emerging area of study (Leite et al., 2013; Fong et al., 2003; Breazeal, 2003), as robots are increasingly finding applications as peers to humans. Although this peerage is not yet on the level of human networks, robots such as smart speakers, mobility assistants and robotic companions for the elderly may be viewed as social robots that take the place of humans in that role.

As AI/ML algorithmic capabilities advance, it is crucial for the social robot to know how to be “human”, to engage in conversations where empathy rather than knowledge is key to establishing trust and equality. A robot who knows everything and wins every game will soon wear out its welcome with children, as will a robot which algorithmically outputs the same information every time. For example, a driver in an unfamiliar location who relies on a navigation robot might ask it for directions every day to the same place. While there is comfort in knowing that the robot never fails and will faithfully offer navigational assistance every time, a human might behave differently by teaching another “how to fish”, instead of procuring the “fish” every time. Such a learning model for the robot goes beyond merely looking up databases to answer a query–it incorporates a more realistic participatory learning model, not unlike one found in human educational environments. In such a participatory learning model, the robot is rewarded for encouraging the human to participate in active problem-solving, instead of providing the answers for every query. Although this form of robot learning is time-consuming and resource-intensive, the process may be incentivized by nudging the robot toward participatory learning models. The data corresponding to such interactions between the human and the robot can be stored on the blockchain, organized in the form of sheaves for analysis and tuning of the parameters in the learning model.

This participatory learning model can also be applied to the scenario of robot-robot interaction. Similar to human environments, robotic environments can be viewed as a hierarchy of heterogeneous robots, possessing different abilities for different environments. A robot that newly joins an environment may be “taught” how to learn the specific circumstances of the environment from other robots. The potential applications of such robots may be found in diverse environments, such as sensing applications or pursuit-evasion games, where the introduction of new robots to an environment might help facilitate richer data gathering and processing abilities. Additional applications could include newer robots introduced in a networked IoT environment, in automation and manufacturing. Further, robots that have to continually upgrade their skillset to “learn” new tasks and social learning from fellow robots might be more conducive to a robust learning experience. The ethical implications of social learning models in robotic environments provide a rich area of study, that is beyond the scope of this paper.

A robot that is embedded with imperfect information and irrationality will be able to self-adjust its level of relating with the human, while constantly learning from its interactions to match the specific environment. This learning mechanism will operate at a cost to the instant gratification on the part of the human, but will ultimately lead to a co-equal model for robots and humans in diverse environments. We expect that, through our computationally efficient sheaf-theoretic model for blockchain applications, robotic interaction in context can be efficiently analyzed to enable robots reciprocate in social environments. The interactions between robots and humans will be stored on the blockchain allowing for distributed analysis and immutability, bound by smart contracts that are constructed with ethical algorithms. The blockchain, in this case, could be envisioned as a sheaf of robot-human and robot-robot interactions classified by task, priority, or any other parameter. The smart contract itself will contain the two parameters of imperfect information and irrationality. The imperfect information parameter will be dynamically adjusted based on the scenario, causing the robot to suppress its dominance, when needed. The irrationality parameter can be chosen to match the environment, such as that of a child, or a human in varying contexts. Of particular importance here is the concept of immutability. For example, in the case of a dispute that leads to disagreements between the human and the robot, not unlike those found between friends or acquaintances, immutability can help “clear the air” and establish what was actually said or performed in a previous setting. It can also serve as a firm record for legal matters to establish accountability for robot actions and human interaction with the robot.

## Social Robots and Blockchain–An Example

Let ${\mathbb{R}}_{\geq 0}$ denote linearly ordered partially ordered set (poset) of non-negative real numbers. The category of continuous intervals Int is composed of objects and morphisms. We call the objects, denoted by 𝑂𝑏 (𝐼𝑛𝑡) ≔ℝ ≥ 0, and denote an object as a single transaction for the blockchain. Given two transactions l and l', the set $Int\left(l,\;l^{'}\right)$ of morphisms between them implies the gluing properties of sheaves, where they collect local data from nearby individual points.

**FIGURE 8:**
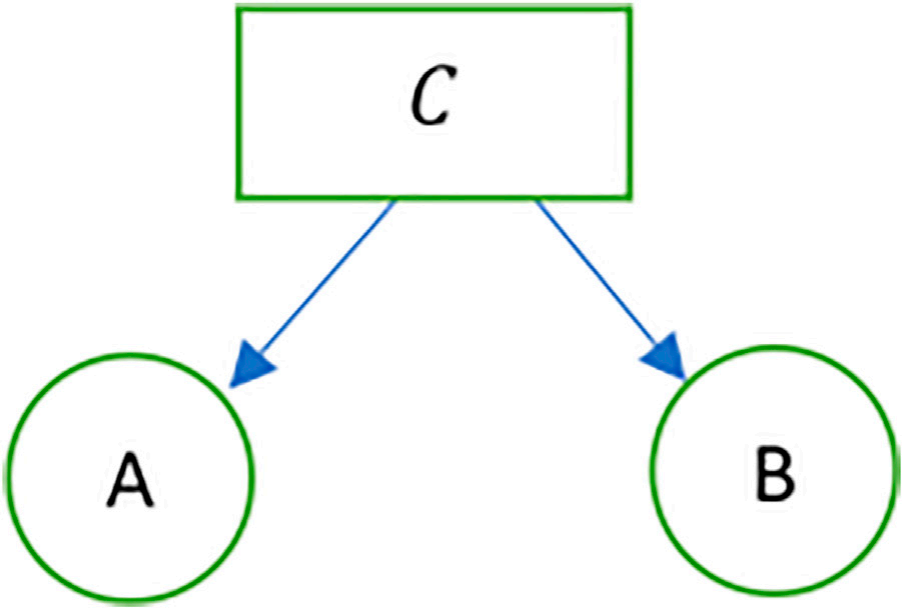
A sheaf representation of transactions.

Let two transactions A and B be defined as A,B ϵ Int. An (A,B) chain is a span in Int represented as follows. The (A, B) chain (Figure 8) is a sheaf C whose sheaf map is given by f:C→A ×B.

### Composition of Blocks and Chains

**FIGURE 9:**
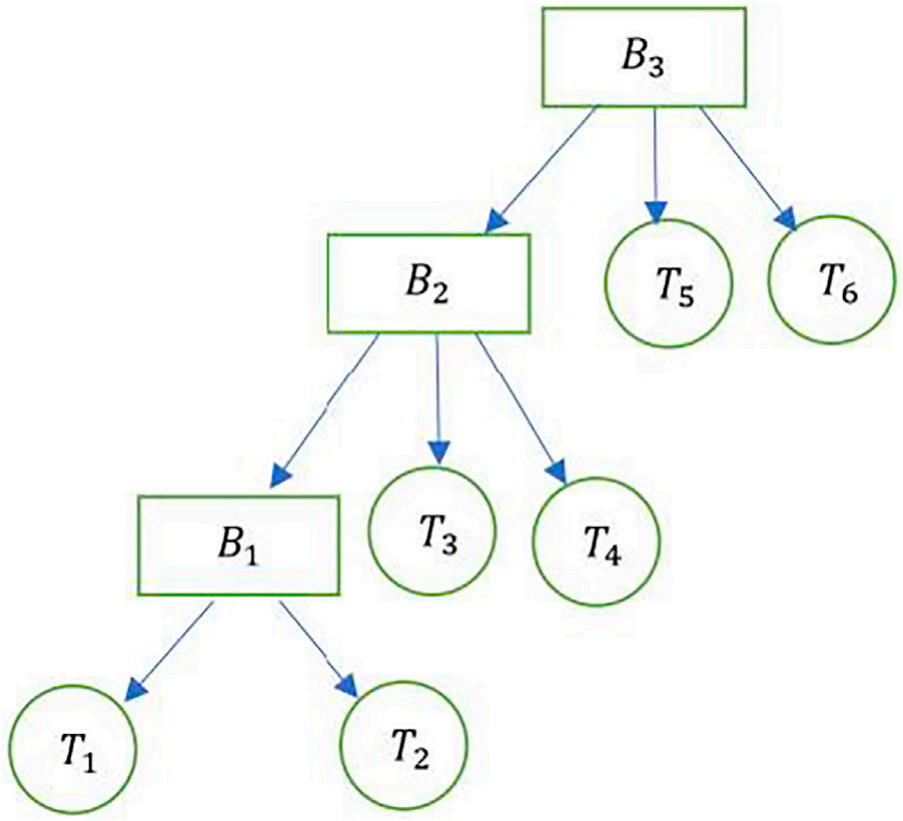
Representing blocks, transactions and hashes as a sheaf.

The Merkle tree-like representation of the blockchain as a sheaf is shown in Figure 9. Consider block $B_{1}$ that contains two transactions, $T_{1}$ and $T_{2}.$ Represented as a sheaf along the lines of Figure 7, transactions $T_{1}$ and $T_{2}$ comprise the inputs to block $B_{1}.$ The output of block $B_{1}$ is the hash of $B_{1}$, and along with transactions $T_{3}$ and $T_{4}\;$forms the set of inputs to block $B_{2}$. This process continues for block $B_{3},$ whose inputs are the transactions $T_{5}$ and $T_{6}$, along with the hash of block. $B_{2}.\;$

**FIGURE 10:**
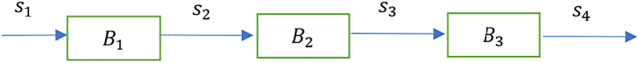
A sheaf containing blocks in series.

The blocks $B_{1},\;B_{2}$ and $B_{3}$ can be represented as a sheaf of blocks in series, as shown in Figure 10. $s_{i}$ denote the set of inputs to block $B_{i}$.

**FIGURE 11:**
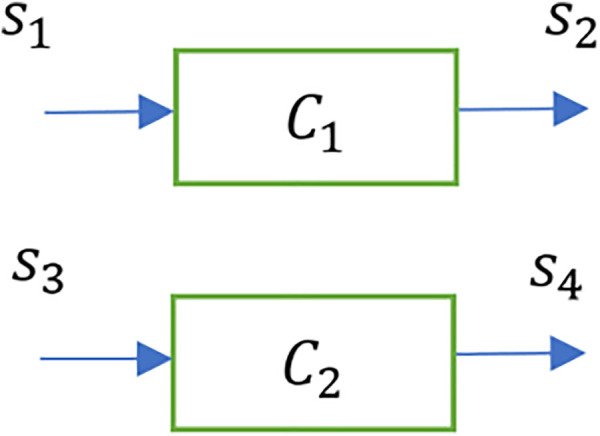
Composition of a sheaf of parallel chains

Multiple chains, each of which is represented by the schematic in Figure 10 can be combined to result in a composition of chains $C_{i},$ as shown in Figure 11. This represents sheaves in parallel, which in the case of Figure 10 denotes chains in parallel. The inputs and outputs of this system of parallel chains are represented by tensor products. Thus, the input and output are respectively given by $s_{1}\times s_{3}$, and $s_{2}\;\times s_{4}$.

### A Social Robot Example: Voice Assistant Robot

**FIGURE 12:**
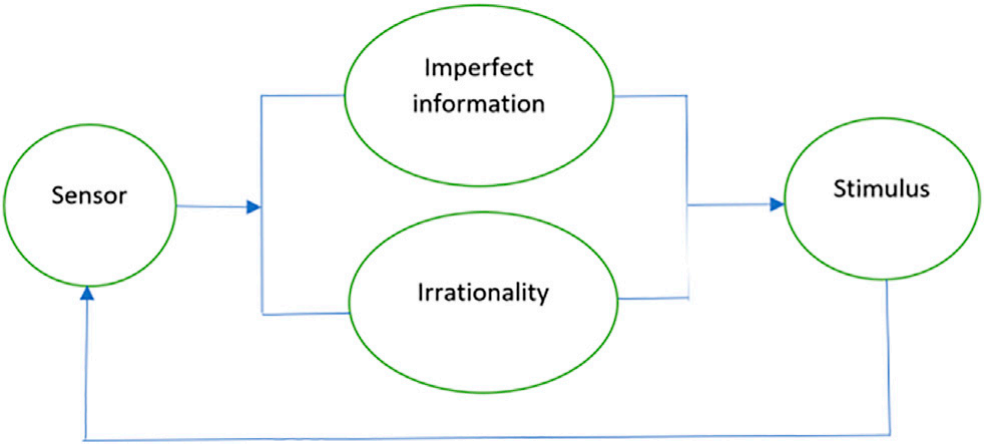
Composition of sheaves in a voice assistant social robot.

In this section, we provide an example of how a voice assistant social robot whose interactions with the environments are stored on the blockchain can be represented with the above-mentioned constructs of sheaf composition. Voice assistant social robots are found in numerous environments ranging from devices like Alexa and Siri to the more sophisticated ones such as Sophia (Weller, 2017). While we exemplify the voice assistant robot, the example can be applied to the case of a multi-sensor robot. Figure 12 provides a representation. The sensor of the voice assistant robot receives a stimulus, which is then tuned with the parameters of imperfect information and irrationality. Each of the four components in the system depicted in Figure 12 can be represented as a sheaf, thus rendering Figure 12 as a composition of sheaves.

**FIGURE 13:**
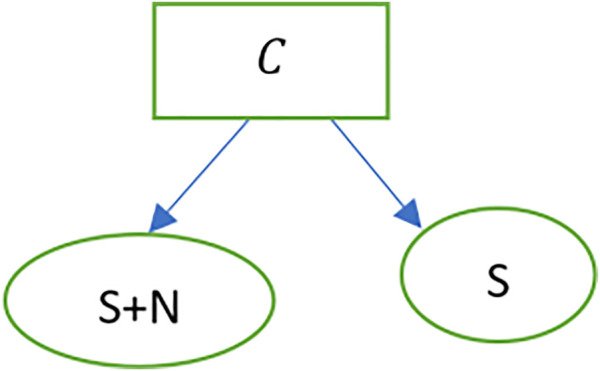
Stimulus sheaf as a blockchain.

Sensor: The sensor updates its goals and beliefs from information about past events in real-time. For an audio stimulus, the social robot will receive the input (S+N), filter out the noise (N) and estimate the meaning of the text. The sheaf is represented as shown in Figure 13.

Stimulus: Let S denote the audio stimulus, which is the set of elements comprising the voice input to the sensor (sequence of words or intelligible vocalizations). The stimulus is the sum $\underset{s\epsilon S}{\sum }M_{s}$, where $M_{s}$ is the additive meaning of a sequence. For example, “Robot X, what is the time?” will be parsed as the meaning of individual words as well as the context of the sentence in the following manner. “Robot–Robot X–Robot X what–Robot X what is–Robot X what is the–Robot X what is the time? The gluing of these sections of the stimulus can be facilitated by the stimulus sheaf. Additionally, by composing a stimulus system made of other attributes such as tone and sentiment (which could themselves be sheaves), further information can be stored on the blockchain sheaf similar to the representations shown in Figures 9–11.

Imperfect information: The information obtained by the sensor presents varying degrees of uncertainty. For the case of the audio input, this uncertainty is captured by the sheaf P, which is composed of attributes such as varying dialects, gender/age specific vocal differences, and a general inability to understand and/or process the text.

Irrationality: The amount of irrationality represented by the robot in its interactions depends on the varying freedoms available to the robot to act outside of its stated goals or beliefs. Humans exhibit irrationality in varying degrees in differing circumstances, however, robots are programmed to work steadfastly toward a goal. The amount of irrationality exhibited by a robot in any interaction can be represented as a sheaf R that is composed of circumstantial affects. For example, a social robot might talk in silly voices with a toddler, answer the toddler’s requests in a jovial manner, while behaving differently with the parents. In this scenario, the social robot’s irrationality can be stored on the blockchain, which can then be represented as a sheaf. Similar to the case of imperfect information, additional information concerning irrationality can be combined to form a composition of chains, all of which represent the sheaf. R. 

## Challenges of Using the Blockchain Sheaf for Robotics Applications

This section describes the challenges and limitations of using the blockchain sheaf for robotics applications. We focus on three aspects of the blockchain sheaf related to diversity of blockchain applications, size of the blockchains and myopic key management tools. First, the use of separate blockchains for different applications and the inability to “smartify” every aspect of human society presents a hindrance to the application of uniform sheaf-theoretic analytic tools. Further, smart contracts that are valid for one application might find limited applicability in other applications, and thus the scope of the jurisdiction in case of arguments becomes contentious as humans seek to find the right entity that is to be held accountable for certain actions. As humans and robots interact over a range of domains, the interaction data may be stored in separate blockchains thereby increasing the complexity of the blockchain networks and the tools to analyze them.

Secondly, scalability of rapidly expanding blockchains is crucial. Blockchain relies on distributed consensus to add new transactions to the block. Copies of the blockchain are stored on several nodes, and the consensus process, although resulting in immutable records, is slow. As robots tune their parameters of imperfect information and irrationality to interact with varying clients, records of their interactions that are stored on the blockchain will have to undergo the same vetting process of approval and distributed consensus. When coupled with the task of correlating information from multiple blockchains, issues of scalability will be paramount. Lastly, discernment of truly private and important information will be a challenging issue in social robotics. Key management of the blockchain sheaf will raise significant challenges in the absence of robotic understanding of what is important. Since, management of critical assets on the blockchain relies heavily on management of private keys, in networks inhabited by robots and humans, imperfect information on the part of both robot and human entities might lead to insufficient protection mechanisms for private keys. While it is relatively easy to train the robot to recognize the value of cryptocurrencies and thereby enforce strict protection mechanisms for private keys controlling wallets, it might be harder for a robot to understand the value of other kinds of data from diverse sources such as IoT devices, supply chain data, smart records, education credentials, and gaming data. While it may be convenient to think that all data is valuable and should be stored on the blockchain, it immediately raises the previously mentioned challenges of information overload on the blockchain. Further, the smart contracts that are embedded with imperfect information and irrationality might not have been encoded with enough foresight, thereby creating myopic smart contracts that do not recognize the value of some data in the present, but later turns out to be valuable data in retrospect.

## Conclusion

In this paper, we proposed a novel framework for visualizing robotics applications on the blockchain that used sheaf theoretic constructs from algebraic topology. To illustrate our framework, we provided examples of robotic blockchain sheaves for various applications. The work in this paper highlights multiple areas for future research in incorporating applied topological constructs to study blockchain applications. This includes studies concerning the structure of blockchain which could use local/global properties of blockchain sheaves to reveal hidden properties of the architectures. Areas of potential research include the revised dynamics of the blockchain with AI/ML applications for specific contexts of human-social interaction.
